# Political instability and supply-side barriers undermine the potential for high participation in HIV testing for the prevention of mother-to-child transmission in Guinea-Bissau: A retrospective cross-sectional study

**DOI:** 10.1371/journal.pone.0199819

**Published:** 2018-08-01

**Authors:** Dlama Nggida Rasmussen, Holger Werner Unger, Morten Bjerregaard-Andersen, David da Silva Té, Noel Vieira, Inés Oliveira, Bo Langhoff Hønge, Sanne Jespersen, Margarida Alfredo Gomes, Peter Aaby, Christian Wejse, Morten Sodemann

**Affiliations:** 1 Center for Global Health, University of Southern Denmark, Odense, Denmark; 2 Department of Infectious Diseases, Odense University Hospital, Odense, Denmark; 3 Bandim Health Project, INDEPTH Network, Bissau, Guinea-Bissau; 4 Department of Obstetrics and Gynaecology, The Royal Infirmary of Edinburgh, Edinburgh, United Kingdom; 5 Department of Medicine at the Doherty Institute, The University of Melbourne, Parkville, Australia; 6 Research Center for Vitamins and Vaccines, Statens Serum Institut, Copenhagen, Denmark; 7 Department of Endocrinology, Hospital of South West Denmark, Esbjerg, Denmark; 8 National HIV Programme, Secretariado Nacional de Luta Contra Sida, Ministry of Health, Bissau, Guinea-Bissau; 9 Association Ceu e Terras, Bissau, Guinea-Bissau; 10 Department of Clinical Immunology, Aarhus University Hospital, Aarhus, Denmark; 11 Department of Infectious Diseases, Aarhus University Hospital, Aarhus, Denmark; 12 Department of Obstetrics, Simão Mendes National Hospital, Bissau, Guinea-Bissau; 13 GloHAU, Center for Global Health, Department of Public Health, Aarhus University, Aarhus, Denmark; The Ohio State University, UNITED STATES

## Abstract

**Background:**

The World Health Organization recommends HIV testing is included in routine screening tests for all pregnant women in order to prevent mother-to-child-transmission of HIV and reduce maternal morbidity and mortality.

**Objectives:**

To assess the proportion of women approached and tested for HIV at delivery and factors associated with non-testing at the maternity ward of the Simão Mendes National Hospital (HNSM) in Bissau, Guinea-Bissau.

**Methods:**

We conducted a retrospective cross-sectional study among women presenting for delivery from June 2008 until May 2013. During the study period, national policy included opt-out HIV-testing at delivery. Modified Poisson regression models were used to examine the association of maternal characteristics with HIV testing. Time trends were determined using Pearson’s χ^2^ test.

**Results:**

Seventy-seven percent (24,217/31,443) of women presenting for delivery were counselled regarding PMTCT, of whom 99.6% (24,107/24,217) proceeded with HIV testing. The provision of opt-out HIV testing at labour increased from 38.1% (1,514/3973) in 2008 to 95.7% (2,021/2,113) in 2013, p<0.001. There were four distinct periods (two or more consecutive calendar months) when less than 50% of women delivering at HNSM were tested. Periods of political instability were significantly associated with not testing for HIV (adjusted prevalence ratio [APR] 1.79; 95% CI 1.73–1.84), as was a lower educational status (APR 1.05; 95% CI 1.00–1.10), admission during evenings/nights (APR 1.05; 95% CI 1.01–1.09) and on Sundays (APR 1.14; 95% CI 1.07–1.22) and Mondays (APR 1.12; 95% CI 1.05–1.19).

**Conclusions:**

Rapid scale-up of PMTCT HIV testing services and high testing coverage was possible in this resource-limited setting but suffered from regular interruptions, most likely because of test stock-outs. Establishing proper stock management systems and back-up plans for periods of political instability is required to ensure the maintenance of health system core functions and increase health system resilience.

## Introduction

Prevention of mother-to-child transmission (PMTCT) of HIV through testing and the provision of antiretroviral therapy (ART) to HIV-positive women has proven an effective strategy to combat the HIV epidemic. In 2014, there were an estimated 220,000 cases of vertical transmission worldwide, which was a decline of 58% when compared to estimates from the year 2000 [1].

A cornerstone of PMTCT is the provision of uninterrupted HIV testing and counselling during pregnancy, as it provides a vital entry point to HIV prevention, treatment, care and support services [2]. However, only 44% of pregnant women in low- and middle-income countries were tested for HIV in 2013 [3] and many women presenting for labour remain unaware of their HIV status [4, 5]. While 76% of women in West and Central Africa accessed skilled antenatal care at least once, only half (52%) received the recommended minimum of four antenatal visits between 2010 and 2015 [6]. Studies have shown that provider initiated (opt-out) HIV testing of pregnant women resulted in a significantly higher coverage, compared with patient-initiated (opt-in) testing [7]. In settings with inadequate antenatal care or poor attendance, opt-out HIV testing at delivery may represent the only opportunity for HIV testing for many women [8].

Numerous supply-side barriers to the optimal uptake of PMTCT occur within the health care system, including distance to testing facilities [9], unskilled antenatal care attendants [10], lack of counselling rooms, inadequate staff training [11], inadequate time or resources to deliver PMTCT interventions and irregular supply of testing kits [12, 13]. HIV-test supply interruptions are likely caused by mechanisms similar to interruptions of HIV-drug supply and other essential health products. These include forecasting supply challenges such natural disasters and political upheaval, suboptimal management of stock information and supply chains and unreliable infrastructure [13–16].

Guinea-Bissau in West Africa has 1.8 million inhabitants, of whom 400,000 reside in the capital Bissau [17]. Since its independence in 1974, the country has experienced significant political upheaval, including a civil war in 1999 and five military coups during the period 2002–2012 [18, 19]. According to the Fragile State Index, Guinea-Bissau is currently ranked number 17 out of 178 nations based on its level of instability [20]. The political instability in the country has impeded development despite national and international efforts [21, 22], and health programmes are frequently suspended or delayed [23, 24].

An estimated 6.7% of the population in Guinea-Bissau is infected with HIV [25]. In 2012, an estimated 5,900 children (<15 years) were living with HIV in the country [26]. HIV testing and treatment has been available at selected antenatal clinics in Bissau since 2002 [27]. However, Guinea-Bissau first engaged in scaling-up antiretroviral therapy in 2005. In 2007, efforts were boosted to increase antenatal counselling, testing, and treatment at public health centres due to further support from the Brazilian Government and the Global Fund to Fight AIDS, Tuberculosis and Malaria [28]. Despite these efforts, a high proportion of women presenting for delivery in the capital had not received antenatal counselling and testing, and thus, the Government initiated opt-out counselling and testing at the main maternity hospital, at the time of delivery, in order to identify HIV-infected women in need of care. Research from Guinea-Bissau has shown that 49% of HIV-infected patients presented with advanced disease (CD4 cell count below 200 cells/μL) and an additional one quarter were late presenters (CD4 cell count below 350 cells/μL), which underlines the need for intensified efforts to identify, enrol and retain HIV-infected individuals into care [29].

The aim of this study was to assess the proportion of women approached and tested for HIV around the time of delivery, as well as factors associated with not testing for HIV, using routine survey data collected throughout a five-year period at the maternity ward at the main National Hospital in Bissau, Guinea-Bissau.

## Materials and methods

### Study setting

This study was conducted within the framework of the Bandim Health Project (BHP) (http://www.bandim.org), at the Simão Mendes National Hospital (HNSM) maternity ward in Bissau, the capital of Guinea-Bissau. This public facility is the principal provider of comprehensive emergency obstetric care in Guinea-Bissau. Opt-out HIV counselling and testing was offered to all women admitted to the maternity ward for the management of labour and delivery, miscarriage, birth before arrival or illness in pregnancy. HIV testing and ART was offered free of charge by the Guinean Ministry of Health, but women were required to pay a flat fee of 1500–2000 FCFA (~3–4 USD) for delivery services.

### Study design and participants

We conducted a retrospective cross-sectional analysis of opt-out HIV testing frequency and associated factors for not testing on data routinely collected through the BHP surveillance system from June 2008 until May 2013. All women presenting at the HNSM maternity ward for delivery or immediate postpartum care were considered eligible for participation. Hospital protocol required counselling and verbal informed consent before HIV testing for all women attending the HNSM maternity ward.

### Data collection

The HNSM maternity ward maintains a data registration system in which basic socio-demographic data and clinical data are collected. Trained research assistants performed daily data cleaning and, by use of case report forms (CRFs), collected supplementary demographic and clinical information. The CRFs include information regarding age, ethnicity, place of residence, years of schooling, civil status, date of admission and discharge, number of previous pregnancies, and pregnancy outcomes. In addition, midwives were trained to complete a short CRF as part of the counselling and testing routine, collecting data on patient-reported HIV testing earlier in the index pregnancy, and knowledge of serostatus and antiretroviral treatment were given when applicable. Midwives were paid an incentive of 500 FCFA (~1 USD) for each patient counselled, a scheme that was specifically set up to improve opt-out testing at delivery.

### HIV testing

HIV screening was performed using the rapid test Determine^®^ HIV-1/2 (Abbott Laboratories, Abbott Park, ILL, USA). To confirm infection and to discriminate between HIV sub-types, whole blood from women with positive and/or inconclusive screening results was subsequently tested with the SD Bioline HIV-1/2 3.0 rapid test (Standard Diagnostics, Kyonggi-do, South Korea). Since June 2012, the rapid test First Response HIV Card 1–2.0 (PMC Medical, Mumbai, India) has also been used for HIV confirmation and HIV type discrimination.

### Statistical methods

Maternity and HIV testing data were entered into password-secured databases (dBase 5.0, data Based Inc, Vestal, NY, USA; Microsoft Access 2007, Microsoft, Redmond, WA, USA), and datasets were merged and analysed using Stata 14.0 (Stata Corporation, College Station, TX, USA). Pearson’s χ^2^ test was initially used to explore differences in demographic and birth-related characteristics by HIV test status (Not tested/Tested). Periods of low testing coverage were defined as when the proportion of women tested fell below 50% during a calendar month given national HIV test stock diaries were unavailable for the study period. To assess factors associated with not testing, univariate and multivariate modified Poisson regression models with robust error variances were used to estimate prevalence ratios and 95% confidence intervals (CIs) [30, 31]. The multivariate analysis was fitted with statistically significant covariates (p<0.05) from the univariate analysis using Wald’s test, as well as the age variable.

To explore the effect of political instability in Guinea-Bissau, based on the assumption that the impact of an event may have a delayed or sustained effect, we generated six dummy variables defining political instability as the period from the date of a major political event and the subsequent weeks. To select the most appropriate duration we performed a sensitivity analysis using estimates of 2, 3, 4, 5, 6 and 7 week durations of political instability. Based on the results of our analysis, political instability in this study was defined as the period from the date of a major political event and the following four weeks. Missing values were excluded from the univariate and multivariate analysis, as no clear patterns of missing values were observed. Trend over time (calendar year) was determined using Pearson’s χ^2^ test. A p-value of <0.05 was considered significant.

### Ethical considerations

The use of Government surveillance maternity data was approved by the National Ethical Committee in Guinea-Bissau (CNES-2010-018), and a separate ethical approval was obtained for the analysis of HIV testing data and its linkage with delivery data (CNES-2011-030). All data were effectively anonymized before they were accessed. Newly diagnosed HIV-infected women were initiated on antiretroviral treatment at birth and follow-up was organised prior to discharge from hospital.

## Results

### Participant characteristics

Between June 2008 and May 2013, a total of 31,443 women presented to the HNSM maternity ward for delivery, immediate postpartum care (born before arrival), or management of pregnancy-related complications. Seventy-seven percent (24,217/31,443) of women were approached and counselled, of whom 99.6% (24,107/24,217) were subsequently tested for HIV (110 women [0.4%] declined the test). The average total HIV prevalence (HIV-1, HIV-2 and dual infections) was 5.1% (1,232/24,107). Among those counselled and tested at delivery, only 46.6% (11,233/24,107) of women reported having been tested earlier in the index pregnancy, of whom 5.0% (566/11,233) were HIV positive. Previous test results for women not counselled at labour (n = 7,226) were unavailable.

The median age of women was 24 years (interquartile range [IQR] 20–29 years). The principle ethnic groups included Balanta (23%), Fula (22%) and Pepel (14%). Twenty-two percent of women (6,957/31,443) had no formal schooling, 54% (16,909/31,443) were in a monogamous marriage, and 37% (11,754/30,866) were primigravid (Table 1).

**Table 1:** Sociodemographic and clinical characteristics of pregnant women.

		Study population N = 31,443	HIV tested		
Variable		n(col%)	No (n = 7,336) n(col%)	Yes (n = 24,107) n(col%)	*p-value*
**Age (years)**					0.171
	13–19	7,442(23.7)	1,787(24.4)	5,655(23.7)	
	20–24	9,450(30.1)	2,235(30.5)	7,215(29.9)	
	25–29	8,072(25.7)	1,834(25.0)	6,238(25.9)	
	30+	6,479(20.6)	1,480(20.2)	4,999(20.7)	
**Marital status**					<0.001
	Single ^A^	9,139(29.1)	2,187(29.8)	6,952(28.8)	
	Married-monogamous	16,909(53.8)	4,338(59.1)	12,571(52.2)	
	Married-polygamous	5,395(17.2)	811(11.1)	4,584(19.0)	
**Schooling (years)**					<0.001
	None	6,957(22.1)	2,005(27.3)	4,952(20.5)	
	1–6 years	7,655(24.4)	1,861(25.4)	5,794(24.0)	
	7–12 years	14,525(46.2)	3,232(44.1)	11,293(46.9)	
	Missing	2,306(7.3)	238(3.2)	2,068(8.6)	
**Ethnic Group**					0.339
	Balanta	7,158(22.8)	1,682(22.9)	5,476(22.7)	
	Bijagos	540(1.7)	104(1.4)	436(1.8)	
	Felupe	478(1.5)	104(1.4)	374(1.5)	
	Fula	6,915(22.0)	1,653(22.5)	5,262(21.8)	
	Mancanha	2,443(7.8)	565(7.7)	1,878(7.8)	
	Mandinga	3,269(10.4)	784(10.7)	2,485(10.3)	
	Manjaco	2,402(7.6)	542(7.4)	1,860(7.7)	
	Mixta	911(2.9)	229(3.1)	682(2.8)	
	Pepel	4,532(14.4)	1,037(14.1)	3,495(14.5)	
	Saracule	319(1.0)	67(0.9)	252(1.5)	
	Others ^B^	2,447(7.8)	561(7.7)	1,886(7.8)	
	Missing	29(0.1)	8(0.1)	21(0.1)	
**Parity** ^**C**^					0.525
	1	11,754(37.4)	2,788(38.0)	8,966(37.2)	
	2	7,200(22.9)	1,666(22.7)	5,534(23.0)	
	≥3	11,866(37.7)	2,730(37.2)	9,136(37.9)	
	Missing	623(2.0)	152(2.1)	471(2.0)	
**Week day of labour**					<0.001
	Sunday	4,547(14.5)	1,142(15.6)	3,405(14.1)	
	Monday	4,565(14.5)	1,187(16.2)	3,378(14.0)	
	Tuesday	4,377(13.9)	976(13.3)	3,401(14.1)	
	Wednesday	4,581(14.6)	1,034(14.1)	3,547(14.7)	
	Thursday	4,417(14.1)	973(13.3)	3,444(14.3)	
	Friday	4,451(14.2)	1,008(13.7)	3,443(14.3)	
	Saturday	4,505(14.3)	1,016(13.9)	3,489(14.5)	
**Time presenting for labour**					<0.001
	08–16	11,011(35.0)	2,441(33.3)	8,570(35.6)	
	17–07	20,102(63.9)	4,803(65.5)	15,299(63.5)	
	Missing	330(1.1)	92(1.3)	238(1.0)	
**Mode of delivery**					0.622
	Caesarean	4,649(14.8)	1,059(14.4)	3,590(14.9)	
	Vaginal	26,753(85.1)	6,267(85.4)	20,486(85.0)	
	Missing	41(0.1)	10(0.1)	31(0.1)	
**Political instability** ^**D**^					<0.001
	No	27,699(88.1)	6,151(83.9)	21,548(89.4)	
	Yes	3,744(11.9)	1,185(16.2)	2,559(10.6)	
**Year of testing**					<0.001
	2008 (June-December)	3,973(12.6)	2,459(33.52)	1,514(6.3)	
	2009	6,704(21.3)	1,903(25.9)	4,801(19.9)	
	2010	6,702(21.3)	168(2.3)	6,534(27.1)	
	2011	6,254(19.9)	2,353(32.1)	3,901(16.2)	
	2012	5,697(18.12)	361(4.9)	5,336(22.1)	
	2013 (January-May)	2,113(6.7)	92(1.25)	2,021(8.4)	
**BHP study area**					0.193
	No	24,899(79.2)	5,862(79.9)	19,037(79.0)	
	Yes	6,543(20.8)	1,474(20.1)	5,067(21.0)	
	Missing	1(0.0)	-	1(100.0)	

**Note.** Abbreviation: BHP, Bandim health project demographic surveillance site.

^A^ Includes divorced and widowed women.

^B^ Ethnic groups or nationalities, each comprising less than 1% of the sample population (Cape Verdean, Senegalese, Guinean (Republic of Guinea), Balanta Mane, Mansoanca, Nalu, and Geba).

^C^ Including index pregnancy.

^D^ Date of political event + 4 weeks.

### Testing coverage

**Fig 1:**
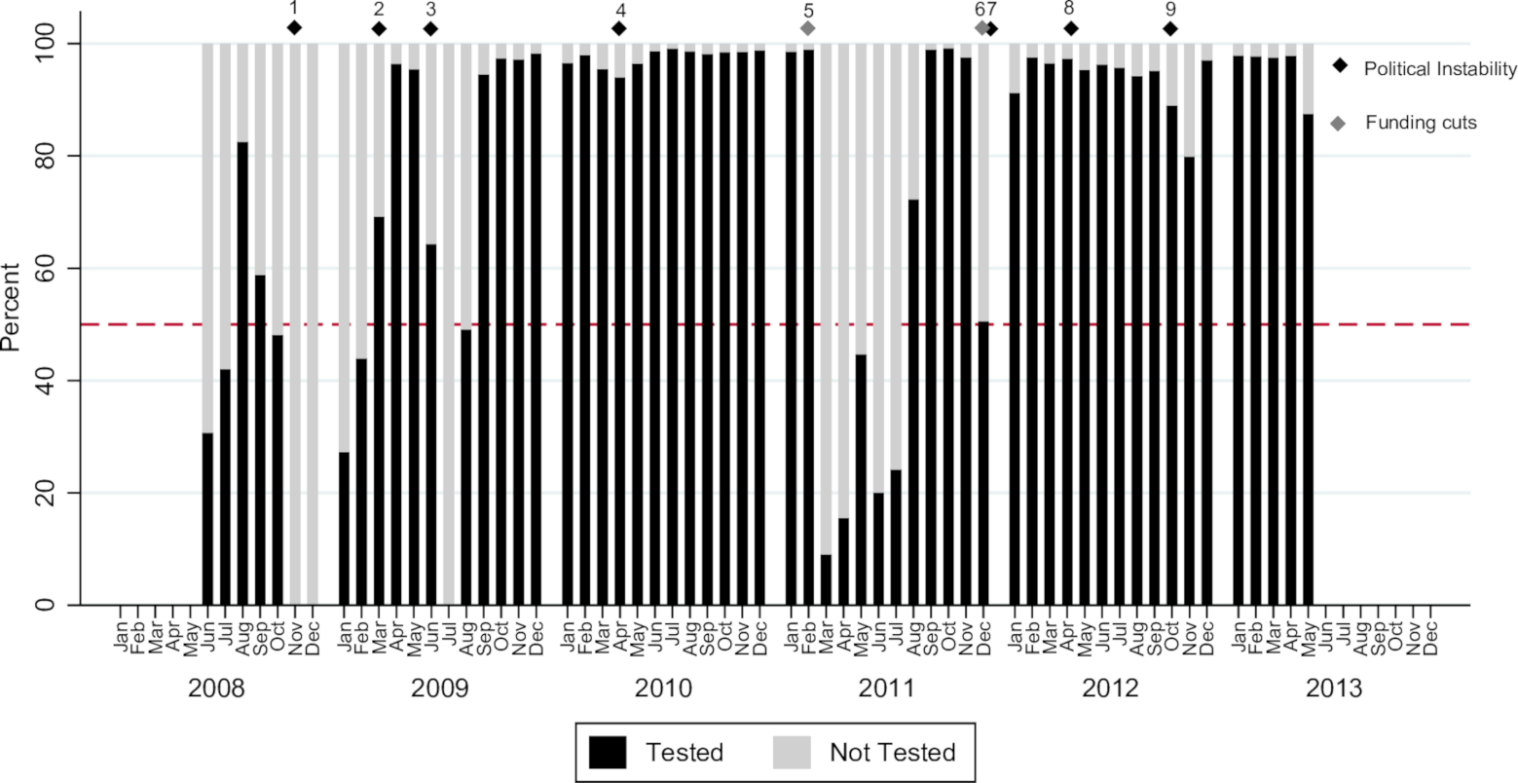
Percent coverage of peripartum opt-out HIV-testing at the Simão Mendes National Hospital, June 2008 to May 2013 (n = 31,443). Legend. The figure displays the percentage of women tested by calendar month and year. The black diamonds indicate periods of political instability, while the grey diamonds indicate major funding cuts. Timeline: 1. Failed coup, gun attack on President Vieira’s home. 2. President Vieira assassinated hours after a bomb attack that killed the army's chief of staff. 3. Military police kill one of the presidential candidates in bid to foil a "coup". 4. Mutinous soldiers detain Prime Minister Carlos Gomes Jr. and replace armed forces chief. 5. EU suspends parts of its aid due to concern over governance. 6. Global Fund (GF) and implementing partners withhold funding to HIV programme. 7. Foiled coup attempt against President Malam Bacai Sanha. 8. Coup-Soldiers topple government, and leading presidential contender Carlos Gomes Jr. arrested. 9. Failed coup attempt.

There were four distinct periods of two or more consecutive calendar months during which less than 50% of women presenting at the HNSM were tested (total of 14 in 60 months): June 2008–July 2008, October 2008-Feburary 2009, July 2009 –August 2009, and March 2011 –July 2011. Two of these periods coincided with major events of political instability and one with major financial instability. During other periods, the average test coverage was 96% (16,499/17,153). The provision of counselling and testing during the study period is displayed in Fig 1.

**Fig 2:**
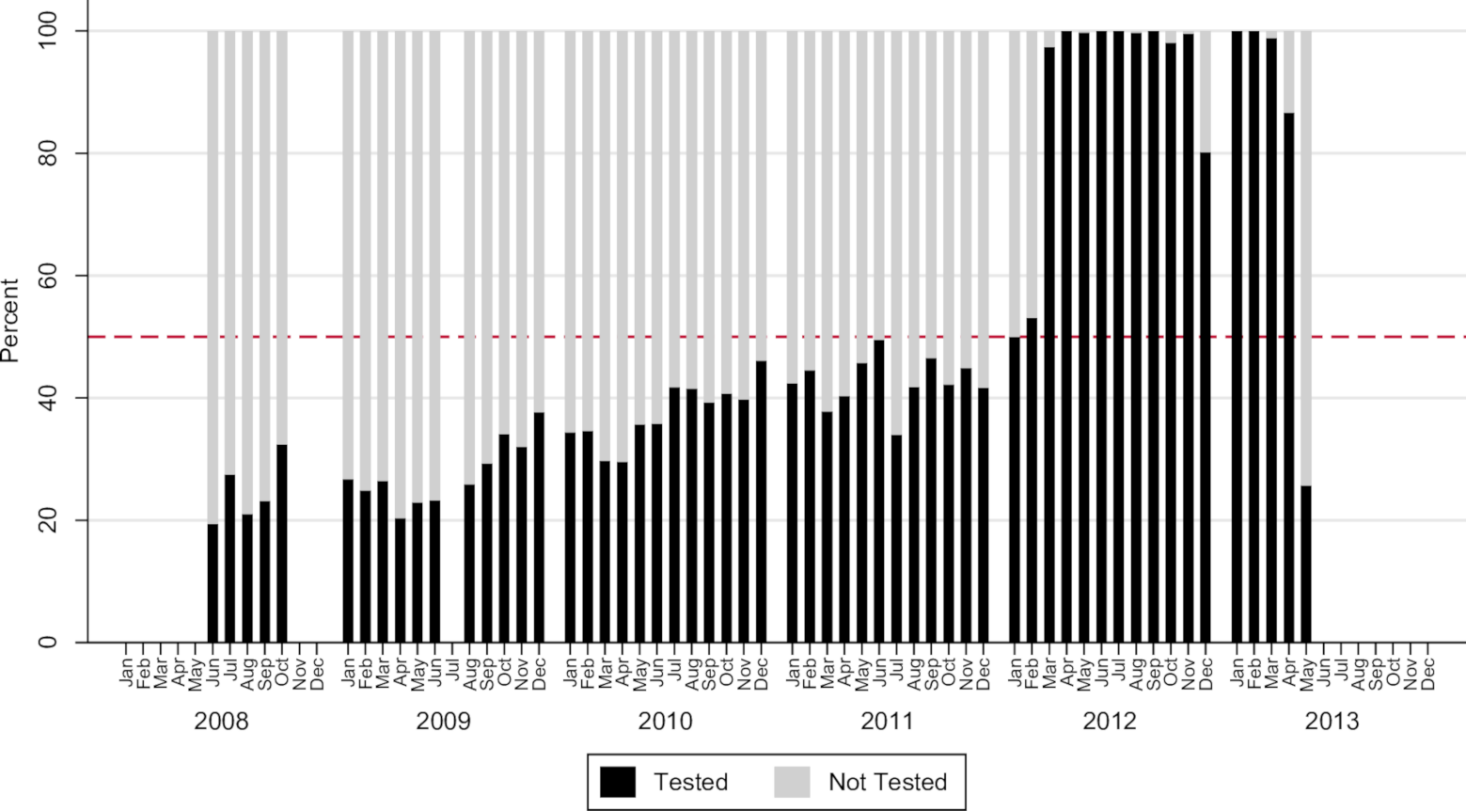
Proportion of women in labour reporting antenatal counselling and testing for HIV, June 2008 to May 2013 (n = 22,458). Legend. The figure displays the percentage of women who reported testing for HIV during antenatal visits by calendar month and year. A notable increase is seen between 2011 and 2012, after which testing declines in May 2013.

Coverage of peripartum opt-out testing and counselling at childbirth increased from 38% (1,514/3973) in 2008 to 96% (2,021/2,113) in the first quarter of 2013, p<0.001. Testing coverage fell from 98% (6,534/6,702) in 2010 to 62% (3,901/6,254) in 2011 before stabilizing again at 94% (5,336/5,697) in 2012. There was a steady increase in the proportion of women testing for HIV antenatally (self-reported), from 25% (354/1,427) in 2008 to 89% (4,384/4,926) in 2012, with a decline to 81% (1,259/1,548) during the first two quarters of 2013, p<0.001 (Fig 2).

### Duration of political instability

We performed a sensitivity analysis to determine the most appropriate duration of political instability. Our results showed an increase in the percentage of women not tested during political instability from 19.5% at 2 weeks to 31.7% at 4 weeks. No significant increase was observed between 4 and 7 weeks (S1 Table). We therefore chose 4 weeks as the appropriate duration of political instability in the univariate and multivariate analysis.

### Factors associated with peripartum HIV testing

In the unadjusted analyses, factors associated with not testing for HIV included lower educational status (primary or below), presenting for labour on Sunday or Monday, and presenting for labour in the evening/night hours (Table 2). Conversely, single women and women in a polygamous marriage (having one or more co-wives) were more likely to test for HIV compared with women in a monogamous marriage. No associations were observed for age, parity or mode of delivery. Political instability was associated with not testing in the unadjusted analyses (crude prevalence ratio [CPR] 1.43; 95% CI 1.35–1.50).

**Table 2:** Crude and adjusted prevalence ratios for not testing for HIV among pregnant women.

Variable		Crude PR (95% CI)	*p-value*	Adjusted PR (95% CI)^E^*	*p-value*
**Age (years)**					
	13–19	1		1	
	20–24	0.99(0.93–1.04)	0.584	1.01(0.97–1.06)	0.616
	25–29	0.95(0.89–1.00)	0.057	1.01(0.96–1.07)	0.603
	30+	0.95(0.90–1.01)	0.105	1.04(0.98–1.10)	0.183
**Marital status**					
	Single ^A^	0.93(0.89–0.98)	0.002	0.99(0.95–1.10)	0.742
	Married-monogamous	1		1	
	Married-polygamous	0.59(0.55–0.63)	<0.001	0.78(0.73–0.83)	<0.001
**Schooling (years)**					
	None	1.30(1.23–1.36)	<0.001	1.05(1.00–1.10)	0.035
	1–6 years	1.09(1.04–1.15)	<0.001	1.06(1.02–1.11)	0.007
	7–12 years	1		1	
	Missing	-			
**Ethnic Group**					
	Balanta	1			
	Bijagos	0.82(0.69–0.98)	0.028		
	Felupe	0.93(0.78–1.10)	0.389		
	Fula	1.02(0.96–1.08)	0.571		
	Mancanha	0.98(0.91–1.07)	0.709		
	Mandinga	1.02(0.95–1.10)	0.589		
	Manjaco	0.96(0.88–1.05)	0.350		
	Mixta	1.07(0.95–1.21)	0.269		
	Pepel	0.97(0.91–1.04)	0.443		
	Saracule	0.89(0.72–1.11)	0.310		
	Others^B^	0.98(0.90–1.06)	0.564		
	Missing	-			
**Parity** ^**C**^					
	1	1			
	2	0.98(0.93–1.03)	0.361		
	≥3	0.97(0.93–1.02)	0.196		
	Missing	-			
**Week day of labour**					
	Sunday	1.11(1.03–1.20)	0.004	1.14(1.07–1.22)	<0.001
	Monday	1.15(1.07–1.24)	<0.001	1.12(1.05–1.19)	0.001
	Tuesday	0.99(0.92–1.07)	0.757	1.04(0.97–1.11)	0.336
	Wednesday	1		1	
	Thursday	0.98(0.90–1.05)	0.536	0.98(0.92–1.05)	0.631
	Friday	1.00(0.93–1.08)	0.932	1.04(0.98–1.12)	0.227
	Saturday	1.00(0.93–1.08)	0.983	1.00(0.93–1.07)	0.905
**Time presenting for labour**					
	08–16	1		1	
	17–07	1.08(1.03–1.13)	0.001	1.05(1.01–1.09)	0.012
	Missing	-			
**Mode of delivery**					
	Caesarean	1			
	Vaginal	1.03(0.97–1.09)	0.338		
	Missing	-			
**Political instability** ^**D**^					
	No	1		1	
	Yes	1.43(1.35–1.50)	<0.001	1.79(1.73–1.84)	<0.001
**Year of testing**					
	2008 (June-December)	1		1	
	2009	045(0.44–0.48)	<0.001	0.51(0.48–0.53)	<0.001
	2010	0.04(0.04–0.05)	<0.001	0.04(0.04–0.05)	<0.001
	2011	0.61(0.58–0.63)	<0.001	0.69(0.66–0.72)	<0.001
	2012	0.10(0.09–0.11)	<0.001	0.11(0.10–0.13)	<0.001
	2013 (January-May)	0.07(0.06–0.09)	<0.001	0.08(0.06–0.10)	<0.001
**BHP study area**					
	No	1.05(0.99–1.10)	0.085		
	Yes	1			
	Missing	-			

**Note.** Abbreviations: PR, prevalence ratio. BHP, Bandim health project demographic surveillance site.

^A^ Includes divorced and widowed women.

^B^ Ethnic groups or nationalities, each comprising less than 1% of the sample population (Cape Verdean, Senegalese, Guinean (Republic of Guinea), Balanta Mane, Mansoanca, Nalu, and Geba).

^C^ Including index pregnancy.

^D^ Date of political event + 4 weeks (not including funding cuts).

^E^ Adjusted prevalence ratio, model including variables with p < 0.05 and age by groups.

*An interaction term for political instability x calendar year is also included.

The multivariate model (n = 28,850) included age, marital status, schooling, political instability, calendar year, weekday and time of labour (Table 2). The adjusted model confirmed univariate findings regarding lower educational status, admission on Sundays or Mondays, and time of labour as significant risk factors for not testing, while polygamy associated with testing. In the adjusted analyses, political instability was also associated with an increased risk of not getting tested (adjusted risk ratio [ARR] 1.79 (95% CI 1.73–1.84)).

## Discussion

The present study assessed the proportion of women approached and tested for HIV at delivery as well as factors associated with HIV testing as a part of PMTCT services at the principal maternity ward in Guinea-Bissau. The data show that the overall provision of self-reported antenatal and opt-out HIV-testing as a part of PMTCT services improved significantly between 2008 and 2013. Nevertheless, almost a quarter of all women were not tested peripartum. While periods with stable, uninterrupted testing with high test uptake were observed, this study suggests that political and financial instability negatively affect the proportion of women tested for HIV.

Our study shows that a rapid scale-up of PMTCT services is possible. Research from other sub-Saharan African countries where opt-out testing is performed has shown that up to 94% of women test for HIV [7]. The low refusal rate in this study may support the high acceptability of opt-out testing observed in other countries [32–36]. However, qualitative research from Guinea-Bissau suggests that testing around the time of delivery may in some instances have been done without adequate informed consent, which may have contributed to the high testing rates observed in this study [37].

We found a steady increase in self-reported antenatal testing, which corresponds to reports by the Guinean HIV programme during the study period [38]. According to national figures from 2014, an estimated 68% of all pregnant women who were tested during antenatal visits received their results [39]. Despite these achievements, repeated periods of non-testing clearly affected the provision of PMTCT. The periods of low and inconsistent testing peripartum observed in this study may have contributed to the high estimated rates of HIV-infected children (22%-27%) born to seropositive mothers between 2011 and 2014 [39].

Interruptions of HIV treatment [28, 40] and test stock-outs have repeatedly been observed in Guinea-Bissau [23, 24, 41]. While the mechanisms of HIV testing (and ART) stock shortages in Guinea-Bissau are complex, the following factors may have played a role in weakening the national HIV response: 1) heavy workload related to preparations of funding proposals leading to delayed implementation of planned activities and subsequently resulting in a failure to meet the goals set by donors [23]; 2) weakness in PMTCT coordination, procurement and supply management [23, 24]; and 3) suspended or delayed institutional and programmatic support/funding [24, 41]. Tudor Car et al. (2013) reported lack of human, material and financial resources (including HIV test kit stock-outs) as key barriers to the implementation of integrated care in developing countries [4]. While the impact of suspended, delayed or ceased funding has serious consequences, the effect may not be immediate. For example, Guinea-Bissau sustained major funding cuts from key donors beginning in February 2011 and through most of 2012, yet provision of HIV testing in this study remained stable during 2012 due to the late arrival of HIV testing kits originally planned for the 2011 calendar year. Conversely, the effect of decreased funding affected other aspects of PMTCT more rapidly. According to UNICEF, national maternal antiretroviral (ART) prophylaxis initiation rates in Guinea-Bissau decreased from 79 to 54 percent between the first and the fourth quarters of 2012 [24]. Further, suspended funding to the national HIV programme in 2012 resulted in a 6-month stock-out of test kits and a marked reduction in HIV treatment coverage, especially in the PMTCT programme, with only 17.4% of affected women benefiting from treatment in 2013 [41]. Better assessment of a health system’s capacity and weaknesses, directing funding and investments towards vulnerable components of the system, and learning from previous crises could prevent future stock-outs. In addition, larger national and local stocks and more frequent and flexible resupply and finance strategies, together empowering and training human resources locally to navigate the administrative work related to funding and stock management, could mitigate stock-outs [42].

We found an association between periods of political instability and decreased HIV testing. Coup attempts in Guinea-Bissau have previously resulted in the shutdown of the country’s largest HIV clinic at HNSM, and vital institutions such as the national laboratory have been closed repeatedly because of work force strikes when salaries were not paid [28]. Countries that experience conflict also experience negative health outcomes [43], yet the association between conflict and healthcare outcomes in relation to HIV remains complex and differs widely depending the aetiology and consequences of the conflicts. Nevertheless, the deterioration of health infrastructure during and after conflict results in a failure to effectively prevent the potential transmission of HIV [44–46]. Periods of political insecurity in developing countries can not only undermine ART programmes and lead to treatment interruptions [47] but also create a chronic state of fragility and poor health system resilience [48]. While political instability may discourage donors to invest, the long-term effects of suspending aid may result in a serious loss of ground in the fight against HIV/AIDS in settings such as Guinea-Bissau [28, 49].

Women in a polygamous marriage were more likely to be tested for HIV. This may reflect selective testing by healthcare workers, in particular when test kits stocks are insufficient to test all women. Polygamous women may be viewed as high risk, yet a recent study among pregnant women reported no difference in the risk of HIV infection according to marital status in our setting [27]. In the context of limited resources, increasing staff education on more reliable risk factors for HIV infection could mitigate the potential issue of unsubstantiated preferential testing. Improvements in registration systems and communication between antenatal clinics and hospitals would help prioritisation in the case of limited stocks.

Similar to other studies, women with fewer years of formal education were less likely test for HIV [10, 50]. This is concerning as lower educational status has been shown to be associated with a higher risk of HIV [51]. Poor HIV-related knowledge has been identified as a potential barrier to treatment adherence in Guinea-Bissau, yet being illiterate in itself did not appear to be a barrier for adherence [49, 52]. Whilst many socio-economic factors may be difficult to overcome, focussed and culturally appropriate interventions to improve HIV literacy in vulnerable populations may increase HIV test uptake and optimise treatment adherence.

We found an association between not testing for HIV on Sundays and Mondays as well as less testing during evenings and nights. Research regarding birth outcomes has described higher perinatal mortality during weekend births [53–55]. This “weekend effect” can extend to Monday, as observed in this study [56]. Previous findings from Bissau demonstrated poorer access to care for children arriving at the emergency room between 7 pm and 7 am [57]. Broader coverage of antenatal testing (and documentation of test results) may reduce the workload for clinical staff out of hours, yet antenatal HIV testing coverage remained suboptimal (68%) in 2014 in Bissau, and only 33% of children born to HIV-positive mothers received ART prophylaxis [39]. HIV counselling and testing for untested women must be embedded firmly in the 24-hour maternity service, through training of frontline healthcare workers, adequate staffing, and locally competitive remuneration.

### Strengths and limitations

This is the first study exploring the provision of HIV testing at labour in Guinea-Bissau. The study is based on data from large sample size of women from all parts of Guinea-Bissau’s capital collected over a 5-year period. Data were collected in a real-life setting and thus reflect the condition of PMTCT services in Bissau throughout a period of political instability and conflict.

Our research has several limitations. First, the study used cross-sectional data collected as part of health surveillance but not specifically for the purpose of this analysis. Furthermore, lack of data on socio-economic status, HIV knowledge and perceived stigma limited our risk factor analysis. Lack of access to national stock diaries to corroborate testing data limited our ability to conclude that stock-outs were the principal causes of test interruptions, although local reports suggest supply issues were the main underlying factor [23, 24, 41]. Antenatal testing was self-reported and may have been influenced by recall bias, fear of stigma or social desirability. HNSM has the country’s largest maternity ward and is located in the capital. Therefore, the utilization of and access to PMTCT services and the stock-out situation of HIV tests in this setting may differ notably compared to other hospitals and clinics in rural areas of Guinea-Bissau limiting our ability to generalize our findings to the entire population.

## Conclusion

This study reveals that rapid scale-up PMTCT HIV testing services is possible even in settings with limited resources and political unease. Nevertheless, HIV programmes such as that in Guinea-Bissau must not only be supported in regards to treatment and testing supplies but also importantly aided in establishing proper management of stocks and back-up plans for periods of political and financial instability. While this study did not include specific data on socioeconomic status it shows an important association between marital status and education with reduced testing uptake which must be taken into consideration in the provision of opt out HIV testing. Strengthening antenatal counselling and testing and insuring proper registration and documentation of HIV testing could reduce the need for testing at delivery and improve overall care in Guinea-Bissau.

## Supporting information

S1 Checklist The STROBE checklist of items for evaluation in reports of observations studies. (DOCX)

S1 Table Crude and adjusted prevalence ratios for not testing for HIV among pregnant women during different durations of political instability. (DOCX)
