# Correction: Political instability and supply-side barriers undermine the potential for high participation in HIV testing for the prevention of mother-to-child transmission in Guinea-Bissau: A retrospective cross-sectional study

**DOI:** 10.1371/journal.pone.0212240

**Published:** 2019-02-07

**Authors:** Dlama Nggida Rasmussen, Holger Werner Unger, Morten Bjerregaard-Andersen, David da Silva Té, Noel Vieira, Inés Oliveira, Bo Langhoff Hønge, Sanne Jespersen, Margarida Alfredo Gomes, Peter Aaby, Christian Wejse, Morten Sodemann

There is an error in the last sentence of the Results section. The correct sentence is: In the adjusted analyses, political instability was also associated with not getting tested (adjusted prevalence ratio [APR] 1.79 (95% CI 1.73–1.84)).
