# Supplementary material for: Political instability and supply-side barriers undermine the potential for high participation in HIV testing for the prevention of mother-to-child transmission in Guinea-Bissau: A retrospective cross-sectional study
Source: PLoS One. 2018 Aug 1;13(8):e0199819. doi: 10.1371/journal.pone.0199819 (PMC6070169; doi:10.1371/journal.pone.0199819)
Supplement: S1 Checklist — (DOCX) [file pone.0199819.s001.docx]

**S1 Checklist. The STROBE checklist of items for evaluation in reports of observations studies.**

Title: Political instability and supply-side barriers undermine the potential for high participation in HIV testing for the prevention of mother-to-child transmission in Guinea-Bissau: A retrospective cross-sectional study

STROBE Statement—checklist of items that should be included in reports of observational studies

|  | Item No. | Recommendation | Page  No. | Relevant text from manuscript |
| --- | --- | --- | --- | --- |
| **Title and abstract** | 1 | (*a*) Indicate the study’s design with a commonly used term in the title or the abstract | 1 | “A retrospective cross-sectional study” |
|  |  | (*b*) Provide in the abstract an informative and balanced summary of what was done and what was found | 2-3 | “Abstract” |
| Introduction | | | |  |
| Background/rationale | 2 | Explain the scientific background and rationale for the investigation being reported | 3-5 | “Introduction” |
| Objectives | 3 | State specific objectives, including any prespecified hypotheses | 5 | “The aim of this study was to assess the proportion of women approached and tested for HIV around the time of delivery, as well as factors associated with not testing for HIV, using routine survey data collected throughout a five-year period at the maternity ward at the main National Hospital in Bissau, Guinea-Bissau.” |
| Methods | | | |  |
| Study design | 4 | Present key elements of study design early in the paper | 6 | “We conducted a retrospective cross-sectional analysis of opt-out HIV testing frequency and associated factors for not testing on data routinely collected through the BHP surveillance system from June 2008 until May 2013.” |
| Setting | 5 | Describe the setting, locations, and relevant dates, including periods of recruitment, exposure, follow-up, and data collection | 5-6 | “This study was conducted within the framework of the Bandim Health Project (BHP) (http://www.bandim.org), at the Simão Mendes National Hospital (HNSM) maternity ward in Bissau, the capital of Guinea-Bissau. This public facility is the principal provider of comprehensive emergency obstetric care in Guinea-Bissau.” &  “..from June 2008 until May 2013. “ |
| Participants | 6 | (*a*) *Cohort study*—Give the eligibility criteria, and the sources and methods of selection of participants. Describe methods of follow-up  *Case-control study*—Give the eligibility criteria, and the sources and methods of case ascertainment and control selection. Give the rationale for the choice of cases and controls  ***Cross-sectional study***—Give the eligibility criteria, and the sources and methods of selection of participants | 6 | “All women presenting at the HNSM maternity ward for delivery or immediate postpartum care were considered eligible for participation.” |
|  |  | (*b*) *Cohort study*—For matched studies, give matching criteria and number of exposed and unexposed  *Case-control study*—For matched studies, give matching criteria and the number of controls per case | - | N/A |
| Variables | 7 | Clearly define all outcomes, exposures, predictors, potential confounders, and effect modifiers. Give diagnostic criteria, if applicable | - | N/A |
| Data sources/ measurement | 8* | For each variable of interest, give sources of data and details of methods of assessment (measurement). Describe comparability of assessment methods if there is more than one group | *-* | N/A |
| Bias | 9 | Describe any efforts to address potential sources of bias | 7-8 | “Statistical methods” |
| Study size | 10 | Explain how the study size was arrived at | - | N/A |

Continued on next page

| Quantitative variables | 11 | Explain how quantitative variables were handled in the analyses. If applicable, describe which groupings were chosen and why | 7 | “To explore the effect of political instability in Guinea-Bissau, based on the assumption that the impact of an event may have a delayed or sustained effect, we generated six dummy variables defining political instability as the period from the date of a major political event and the subsequent weeks. To select the most appropriate duration we performed a sensitivity analysis using estimates of 2, 3, 4, 5, 6 and 7 week durations of political instability. Based on the results of our analysis, political instability in this study was defined as the period from the date of a major political event and the following four weeks.” |
| --- | --- | --- | --- | --- |
| Statistical methods | 12 | (*a*) Describe all statistical methods, including those used to control for confounding | 7-8 | “Pearson’s χ2 test was initially used to explore differences in demographic and birth-related characteristics by HIV test status (Not tested/Tested).” &  “To assess factors associated with not testing, univariate and **multivariate modified Poisson regression models with robust error variances were used to estimate prevalence ratios and 95% confidence intervals (CIs)** [30, 31]. The multivariate analysis was fitted with statistically significant covariates (p<0.05) from the univariate analysis using Wald’s test, as well as the age variable..” &  “Trend over time (calendar year) was determined using Pearson’s χ2 test. A p-value of <0.05 was considered significant.” |
|  |  | (*b*) Describe any methods used to examine subgroups and interactions | - |  |
|  |  | (*c*) Explain how missing data were addressed | 8 | “Missing values were excluded from the univariate and multivariate analysis, as no clear patterns of missing values were observed.” |
|  |  | (*d*) *Cohort study*—If applicable, explain how loss to follow-up was addressed  *Case-control study*—If applicable, explain how matching of cases and controls was addressed  *Cross-sectional study*—If applicable, describe analytical methods taking account of sampling strategy |  |  |
|  |  | (*e*) Describe any sensitivity analyses |  |  |
| Results | | | | |
| Participants | 13* | (a) Report numbers of individuals at each stage of study—eg numbers potentially eligible, examined for eligibility, confirmed eligible, included in the study, completing follow-up, and analysed | 9 | “Participant characteristics” |
|  |  | (b) Give reasons for non-participation at each stage | 9 | “Previous test results for women not counselled at labour (n=7,226) were unavailable.” |
|  |  | (c) Consider use of a flow diagram | - | N/A |
| Descriptive data | 14* | (a) Give characteristics of study participants (eg demographic, clinical, social) and information on exposures and potential confounders | 9-10 | “Table 1” |
|  |  | (b) Indicate number of participants with missing data for each variable of interest | 9 | “Table 1. Sociodemographic and clinical characteristics of pregnant women.” |
|  |  | (c) *Cohort study*—Summarise follow-up time (eg, average and total amount) | - | Not relevant |
| Outcome data | 15* | *Cohort study*—Report numbers of outcome events or summary measures over time | *-* | Not relevant |
|  |  | *Case-control study—*Report numbers in each exposure category, or summary measures of exposure | *-* | Not relevant |
|  |  | *Cross-sectional study—*Report numbers of outcome events or summary measures | *9* | “Seventy-seven percent (24,217/31,443) of women were approached and counselled, of whom 99.6% (24,107/24,217) were subsequently tested for HIV (110 women [0.4%] refused the test).” &  “…the average test coverage was 96% (16,499/17,153). The provision of counselling and testing during the study period is displayed in Fig 1. |
| Main results | 16 | (*a*) Give unadjusted estimates and, if applicable, confounder-adjusted estimates and their precision (eg, 95% confidence interval). Make clear which confounders were adjusted for and why they were included | 13-14 | “Table 2. Crude and adjusted prevalence ratios for not testing for HIV among pregnant women..” &  “Adjusted prevalence ratio, model including variables with p < 0.05 and age by groups. *An interaction term for political instability x calendar year is also included.” |
|  |  | (*b*) Report category boundaries when continuous variables were categorized | - | N/A |
|  |  | (*c*) If relevant, consider translating estimates of relative risk into absolute risk for a meaningful time period | - | N/A |

Continued on next page

| Other analyses | 17 | Report other analyses done—eg analyses of subgroups and interactions, and sensitivity analyses | - | N/A |
| --- | --- | --- | --- | --- |
| Discussion | | | | |
| Key results | 18 | Summarise key results with reference to study objectives | 14-15 | “The present study assessed the proportion of women approached and tested for HIV at delivery as well as factors associated with HIV testing as a part of PMTCT services at the principal maternity ward in Guinea-Bissau. The data show that the overall provision of self-reported antenatal and opt-out HIV-testing as a part of PMTCT services improved significantly between 2008 and 2013…” |
| Limitations | 19 | Discuss limitations of the study, taking into account sources of potential bias or imprecision. Discuss both direction and magnitude of any potential bias | 19 | “Our research has several limitations. First, the study used cross-sectional data collected as part of health surveillance but not specifically for the purpose of this analysis. Furthermore, lack of data on socio-economic status, HIV knowledge and perceived stigma limited our risk factor analysis. Lack of access to national stock diaries to corroborate testing data limited our ability to conclude that stock-outs were the principal causes of test interruptions, although local reports suggest supply issues were the main underlying factor…” |
| Interpretation | 20 | Give a cautious overall interpretation of results considering objectives, limitations, multiplicity of analyses, results from similar studies, and other relevant evidence | 18-19 | “Strengths and limitations” |
| Generalisability | 21 | Discuss the generalisability (external validity) of the study results | 14-18 | “Discussion” |
| Other information | |  | | |
| Funding | 22 | Give the source of funding and the role of the funders for the present study and, if applicable, for the original study on which the present article is based | 20 | “This work was supported by the Albert McKern Bequest, Edinburgh, UK, and the Department of Infectious Diseases, Odense University Hospital Denmark.” |

*Give information separately for cases and controls in case-control studies and, if applicable, for exposed and unexposed groups in cohort and cross-sectional studies.

**Note:** An Explanation and Elaboration article discusses each checklist item and gives methodological background and published examples of transparent reporting. The STROBE checklist is best used in conjunction with this article (freely available on the Web sites of PLoS Medicine at http://www.plosmedicine.org/, Annals of Internal Medicine at http://www.annals.org/, and Epidemiology at http://www.epidem.com/). Information on the STROBE Initiative is available at www.strobe-statement.org.
