# Supplementary material for: Political instability and supply-side barriers undermine the potential for high participation in HIV testing for the prevention of mother-to-child transmission in Guinea-Bissau: A retrospective cross-sectional study
Source: PLoS One. 2018 Aug 1;13(8):e0199819. doi: 10.1371/journal.pone.0199819 (PMC6070169; doi:10.1371/journal.pone.0199819)
Supplement: S1 Table — (DOCX) [file pone.0199819.s002.docx]

**S1 Table. Crude and adjusted prevalence ratios for not testing for HIV among pregnant women during different durations of political instability.**

| **Duration of**  **political instability ^A^** | | **HIV tested during political instability** | | **Crude**  **PR (95% CI)** | ***p-value*** | **Adjusted**  **PR (95% CI)^B^** | ***p-value*** |
| --- | --- | --- | --- | --- | --- | --- | --- |
|  |  | **No**  **n(row%)** | **Yes**  **n(row%)** |  |  |  |  |
|  | 2 weeks | 700(19.5) | 2,897(80.5) | 0.82(0.76-0.88) | <0.001 | 1.70(1.65-1.74) | <0.001 |
|  | 3 weeks | 918(27.7) | 2,394(72.3) | 1.22(1.15-1.29) | <0.001 | 1.73(1.69-1.79) | <0.001 |
|  | **4 weeks** | **1,185(31.7)** | **2,559(68.3)** | **1.43(1.35-1.50)** | **<0.001** | **1.79(1.73-1.84)** | **<0.001** |
|  | 5 weeks | 1,483(32.4) | 3,099(67.6) | 1.49(1.42-1.56) | <0.001 | 1.84(1.78-1.90) | <0.001 |
|  | 6 weeks | 1,724(32.2) | 3,621(67.8) | 1.50(1.43-1.57) | <0.001 | 1.91(1.85-1.96) | <0.001 |
|  | 7 weeks | 1,979(32.0) | 4,199(68.0) | 1.51(1.45-1.58) | <0.001 | 1.99(1.92-2.07) | <0.001 |

Note. Abbreviations PR, prevalence ratio; CI, confidence intervals. ^A^ The duration period of political instability was calculated as the date of a political event + a defined number of weeks.  ^B^ Adjusted for age, marital status, years of schooling, weekday of labour, time presenting for labour, year of testing (including an interaction term between for political instability x calendar year.
